# Effects of Cannabidiol on Behavior and Oxidative Stress in a Rat Model of Depression Under Chronic Stress

**DOI:** 10.3390/jox16030073

**Published:** 2026-04-26

**Authors:** George Jîtcă, László-István Bába, Ingrid Evelin Mehelean, Ana Natalia Maier, Ioana-Irina Popoviciu, Tudor-Nicolae Cotruş, Erzsébet Májai

**Affiliations:** 1 Department of Pharmacology and Clinical Pharmacy, Faculty of Pharmacy, George Emil Palade University of Medicine, Pharmacy, Science and Technology of Târgu Mureș, 540139 Târgu Mureș, Romania; george.jitca@umfst.ro; 2 Faculty of Pharmacy, George Emil Palade University of Medicine, Pharmacy, Science and Technology of Târgu Mureș, 540139 Târgu Mureș, Romania; inghridmehelean@gmail.com (I.E.M.); ana.natalia.maier@gmail.com (A.N.M.); irinapopoviciu@gmail.com (I.-I.P.); tudorcotrus@yahoo.com (T.-N.C.); 3 Department of Toxicology and Biopharmacy, Faculty of Pharmacy, George Emil Palade University of Medicine, Pharmacy, Science and Technology of Târgu Mureș, 540139 Târgu Mureș, Romania; erzsebet.fogarasi@umfst.ro

**Keywords:** alcohol, cannabidiol, oxidative stress, behavior, memory

## Abstract

According to the most recent data published by the World Health Organization (WHO), it is estimated that approximately 332 million persons worldwide suffer from depression. The relationship between depression and alcohol consumption is complex and bidirectional. This study aimed to investigate the effects of cannabidiol (CBD) on behavior and malondialdehyde (MDA) imbalance in female Wistar rats exposed to chronic stress and alcohol. Sixteen intact cycle female 5-month-old Wistar rats were randomly assigned to two groups: the Control group (*n* = 8), and the CBD group (*n* = 8), which received CBD at a dose of 10 mg/kg. Following chronic stress induction, during the three-week treatment period, the animals were exposed to alcohol on three separate occasions. CBD-treated females showed increased freezing time in the Open Field test with no clear anxiolytic effect. In the Y maze and Morris Water Maze, they exhibited improved memory-related performance. Brain MDA levels were reduced, while plasma MDA was unchanged. Cortisol tended to be higher in the CBD group. CBD administration showed potential cognitive and central antioxidant effects, but no clear anxiolytic effect.

## 1. Introduction

According to the most recent data published by the World Health Organization (WHO), it is estimated that approximately 332 million people worldwide suffer from depression, which is around 4% of the global population. This prevalence is high among adults; approximately 6% are affected, with a higher incidence among women (7%) compared to men (4.6%) [1]. It is worth mentioning that in the wake of the COVID-19 pandemic, a significant increase in the prevalence of anxiety and depression disorders was observed, with an estimated percentage of 25%. These data highlight the importance of continuing the efforts to raise awareness, prevention and treatment of depression, given its impact on public health worldwide. A common behavior associated with depression is alcohol consumption, which is often used as a coping mechanism to alleviate negative symptoms [1]. The relationship between depression and alcohol consumption is complex and bidirectional, and is supported by clinical and experimental data showing that each of them may constitute a risk factor for the occurrence of the other [2]. Depression is a highly prevalent mental health disorder that has a significant impact on quality of life and daily functioning [1]. Numerous clinical and preclinical studies have shown a strong association between depressive symptoms and problematic alcohol use. This relationship is often bidirectional: on one hand, individuals with depression may use alcohol as a form of self-medication; on the other hand, chronic alcohol consumption may contribute to the onset or worsening of depressive symptoms [2,3].

Preclinical studies might offer significant insight into and mechanistic details about these interactions. Thus, in an animal model performed on FH/WJD rats, a model of vulnerability to depression showed increased alcohol consumption compared to other breeds, suggesting a behavioral predisposition that links depression to addiction [3]. Both depression and alcoholism are associated with redox imbalances and increased levels or reactive oxygen species (ROS), which may disrupt the neuronal structure and function, particularly in hippocampal and prefrontal regions [4]. In addition, alcohol chronic consumption exacerbates this imbalance by disrupting mitochondrial function and decreasing the antioxidant capacity of the central nervous system (CNS) [5].

Associated with redox imbalance, dysregulation of hypothalamic–pituitary–adrenal (HPA) axis might also occur. Loss of mechanisms regulating cortisol secretion are frequently observed in both depression and alcohol use disorders [6]. Hyperactivity of HPA axis leads to a chronic stress response that can promote neuroinflammation, impairment of synaptic plasticity, and depressive or impulsive behaviors [7].

Moreover, a study on C57BL/6J mice showed that acute ethanol administration in 1.75 and 2.25 g/kg doses selectively affects spatial memory, as assessed by the Morris water maze test, without a significant influence on non-spatial memory. This suggests a specific vulnerability of spatial memory to the acute effects of alcohol [8]. Another study investigated the effects of alcohol consumption (both chronic and acute) on the spatial and non-spatial memory of rats. The results showed that both chronic and acute alcohol consumption impair performances with regard to object recognition and spatial recognition, with greater deficits in the case of chronic use [9]. A study examined the impact of alcohol and stress on memory in female rats. The results showed that alcohol—alone or in combination with stress—negatively affects recognition memory and spatial memory, highlighting gender differences in response to these stimuli [10].

In this context, cannabidiol (CBD) gained attention from researchers due to its neuroprotective and psychomodulatory potential. CBD has demonstrated antidepressant, anxiolytic, and alcohol-reducing consumption effects in preclinical studies, through 5-HT1A receptors and the endocannabinoid system [11]. In addition, it has been observed that CBD administration reduces voluntary alcohol consumption and relapse behavior in previously exposed animals [12], as well as cortisol levels and markers of oxidative stress [13].

Thus, CBD emerges as a promising therapeutic option for managing the comorbidity between depression and alcohol use disorders, simultaneously addressing neurochemical, endocrinological and redox imbalances.

Thus, in the present study, we aimed to investigate the effects of CBD on behavior and redox imbalance in female Wistar rats exposed to chronic stress and alcohol.

## 2. Materials and Methods

### 2.1. Experimental Design, Drug Administrations and Animal Selection

The present study utilized 16 intact cycle female Wistar rats aged 5 months, with body weights ranging from 300 to 400 g. The animals were obtained from the vivarium of UMFST George Emil Palade, Târgu Mureș. Group allocation was determined by a computer-generated randomization protocol. No animals were removed from the study as no exclusion criteria were defined prior to or during the experimental period. Before the onset of the experiment, all animals underwent a one-week habituation phase, during which they were handled daily to minimize stress responses. Each animal was housed in an individual cage throughout the study. Subjects were divided into two experimental groups of equal size. The Control group (*n* = 8) received the vehicle substance incorporated into food at 1 mL/kg, while the CBD-treated group (*n* = 8) was administered cannabidiol at a dose of 10 mg/kg mixed into food (1 mL/kg) for a three-week period. Briefly, animals were trained to consume a drug-containing pellet that was offered individually (approx. 0.1 g). This was made of standard laboratory chow and a standardized CBD solution (1 mg/mL in sunflower oil). This way, we omitted the stress of forced administration (i.e., gavage or i.p injection) that could potentially have an impact on our results.

Prior to treatment administration, the animals were subjected to a series of stressors to induce chronic stress. For a period of three weeks, the rats were exposed to various stressors applied on randomly selected days. These included: cage tilting at a 45-degree angle for 24 h (days −20, −15, −11, −4), removal of bedding for 24 h (days −16, −8, −1), cage switching for 24 h (day −3), exposure to a bright light beam for 1 min (days −18, −15, −10, −4, −1), and deprivation of food (days −15, −8, −1) or water (days −17, −12, −6, −2) for 24 h.

**Figure 1:**
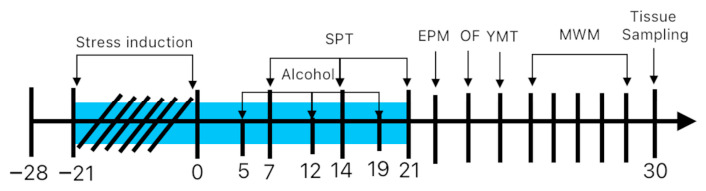
Schematic representation of the study protocol. The subjects underwent psychosocial stress between days −21 and 0. The stressors included the following: cage tilting at a 45-degree angle for 24 h (days −20, −15, −11, −4), removal of bedding for 24 h (days −16, −8, −1), cage switching for 24 h (day −3), exposure to a bright light beam for 1 min (days −18, −15, −10, −4, −1), and deprivation of food (days −15, −8, −1) or water (days −17, −12, −6, −2) for 24 h. The subjects underwent CBD and vehicle treatment, respectively, for 30 days. Alcohol exposure—days 5, 12, 19. Sucrose preference test (SPT) —days 7, 14, 21. EPM, elevated plus maze—day 22; OF, open field—day 23; YMT, Y maze test—day 24; MWM, Morris water maze—days 25–29; tissue sampling—day 30.

A visual representation of the experimental design and timeline is illustrated in Figure 1. Throughout the study, animals were kept in controlled vivarium conditions, including 12 h light/dark, ambient temperature 20 ± 2 °C, and relative humidity kept between 40% and 60%. Food and water were available ad libitum, with the exception of scheduled deprivation intervals applied as part of the chronic stress protocol. Body weight was monitored on a weekly basis to allow appropriate recalculation of individual treatment doses. The study received ethical clearance from the Ethics Committee for Scientific Research of UMFST George Emil Palade, Târgu Mureș (approval number 3325/29.07.2024).

### 2.2. Drugs and Reagents

Cannabidiol (CBD; 99.5% purity, Trigal Pharma GmbH, Vienna, Austria) was prepared by dissolving it in sunflower oil, then mixing it into standard laboratory chow pellet. The prepared food was administered once daily at 12:00. Chromatographic analyses employed the following reagents: acetonitrile (VWR International, Rosny-sous-Bois, France), 85% phosphoric acid and ultrapure water (Merck Millipore Corporation, Burlington, MA, USA), 98% thiobarbituric acid (TBA), phosphate-buffered solution, 99% trimethylpropane (TMP), and 98% sulfuric acid (Chemical Company, Iași, Romania). The analytical methods used for the determination of MDA levels were previously published [14].

### 2.3. Behavioral Tests

The behavioral testing was designed to evaluate two primary domains: anxiety-related responses and cognitive performance. No blinding procedures were applied during the behavioral assessments.

#### 2.3.1. Sucrose Preference Test (SPT) and Alcohol Preference

Following chronic stress induction, during the three-week treatment period, the animals were exposed to alcohol on three separate occasions. During each 24 h exposure session, animals were simultaneously presented with two drinking bottles, one containing water and the other containing a 20% alcohol solution. The positions of the two bottles were alternated at every session to preclude the development of a side preference.

Anhedonic behavior was evaluated using the sucrose preference test. For a period of 24 h, each animal had concurrent access to two bottles, one filled with water and the other with a 1% sucrose solution, with bottle positions counterbalanced between sessions. At the end of the 24 h period, the volume consumed from each bottle was recorded, and the sucrose preference was calculated.

$$\%\mathrm{sucrose}\;\mathrm{preference}=\frac{\mathrm{volume}\;\mathrm{of}\;\mathrm{sucrose}\;\mathrm{solution}\;\mathrm{intake}}{\mathrm{total}\;\mathrm{volume}\;\mathrm{intake}}\times 100$$

$$\%\mathrm{alcohol}\;\mathrm{preference}=\frac{\mathrm{volume}\;\mathrm{of}\;\mathrm{alcohol}\;\mathrm{solution}\;\mathrm{intake}}{\mathrm{total}\;\mathrm{volume}\;\mathrm{intake}}\times 100$$

Sucrose preference (%) is the ratio between the volume of sucrose intake and total volume intake (sum of volume of sucrose solution and volume of water) multiplied by 100. Alcohol preference (%) is the ratio between the volume of alcohol intake and total volume intake (sum of volume of alcohol solution and volume of water) multiplied by 100.

#### 2.3.2. Open Field Test (OF)

General locomotor activity and anxiety-related behavior were assessed using the OF test, which evaluated spontaneous exploratory behavior based on movement patterns and spatial distribution within a predefined arena. Each animal was placed individually at the center of a square enclosure (60 × 60 × 50 cm) and allowed to freely explore for a 5 min session. Behavioral activity was recorded and analyzed using EthoVision XT software (v.11.5, Noldus IT, Wageningen, The Netherlands). The apparatus was thoroughly cleaned with 70% ethanol between consecutive trials to eliminate olfactory cues. Behavioral parameters recorded included distance, time in central zone (30 × 30 cm), frequency in center, and freezing time. Locomotor activity was assessed by measuring the total distance traveled and anxiety was evaluated based on time spent in the center zone, frequency of entries into the center zone and freezing duration.

#### 2.3.3. Y Maze Test (YM)

Spatial working memory was evaluated using the YM test for measuring short-term memory through spontaneous alternation behavior. The test exploits the innate tendency of rodents to preferentially investigate novel or previously unvisited environments. The apparatus consists of an opaque Y-shaped maze comprising three arms (50 × 16 × 36 cm) arranged symmetrically at 120° relative to one another. Each rat was introduced into the maze and permitted to explore all three arms freely for an 8 min session. A valid arm entry was counted only when the animal had placed all four paws within a given arm. Spontaneous alternation was defined as the successive entry into each of the three distinct arms without immediate repetition, serving as in index of intact spatial working memory. After each rat, the maze was cleaned with 70% alcohol. Behavioral parameters recorded included distance, zone alternations, % Y zone alternations, time in zones, and freezing time.

$$\%Y\;zone\;alternations=\frac{number\;of\;correct\;zone\;alternations}{total\;arm\;entries-2}\times 100$$

#### 2.3.4. Elevated Plus Maze Test (EPM)

Anxiety-like exploratory behavior was further evaluated using the EPM test. The apparatus comprised a plus-shaped platform elevated 60 cm above the ground, featuring two opposing arms (50 × 10 cm) and two enclosed arms (50 × 10 × 40 cm). At the start of each trial, the animal was positioned at the center of the maze, oriented toward one of the open arms, and left to explore freely for 5 min. Again, to prevent the influence of olfactory cues from preceding subjects, the maze was wiped down with 70% ethanol after each trial. The following behavioral parameters were recorded and analyzed: time spent in the open arms, closed arms, and central area, along with the frequency of rearing and head-dipping.

#### 2.3.5. Morris Water Maze (MWM)

Spatial learning and memory were evaluated by means of the MWM. The apparatus consisted of a circular tank (90 cm diameter, 60 cm deep) filled with water, within which a circular escape platform (9 cm diameter) was submerged approximately 1 cm beneath the water surface and positioned within a randomly designated quadrant. The pool was conceptually divided into four equal parts (quadrants) and a set of distinct visual landmarks was arranged around the testing room to provide spatial reference cues for navigation.

The acquisition phase (learning) spanned four consecutive days, during which each animal performed four trials per day in order to locate the hidden platform. Individual trials had a maximum duration of 120 s, separated by a 60 s inter-trial intervals. The starting point for MWM varied randomly across trials. Animals that failed to locate the platform within the allotted time were gently guided to it and permitted to remain there for 60 s to reinforce spatial learning.

On the fifth day, the trial was carried out in the absence of the escape platform. Each rat was released from the quadrant diametrically opposite to the former platform location and allowed to swim freely for 60 s. The proportion of the time spent in the target quadrant and the latency to reach the former platform position were recorded as primary indices of spatial memory retention.

### 2.4. Plasma and Brain Sampling

Blood sampling was performed under isoflurane anesthesia (3%). During the anesthesia 0.5 mL of peripheral blood was collected from the tail vein into K3 EDTA-coated collection tubes and centrifuged at 3500 rpm for 10 min at 4 °C to collect plasma that was used throughout all biochemical assessments. The final blood collection was performed via intracardiac puncture, and plasma was isolated using the same centrifugation protocol. Following decapitation, brain tissues were harvested, rinsed with phosphate buffer, and weighed before being snap-frozen in liquid nitrogen. All plasma and brain samples were stored at −80 °C.

#### 2.4.1. Oxidative Stress Parameters

Brain samples designated for MDA quantification were homogenized (5 min) using IKA Ultra-Turrax Drive (Königswinter, Germany). Proteins were precipitated with acetonitrile, followed by centrifugation (10,000× *g*, 10 min). The resulting supernatant was diluted with distilled water. For the colorimetric reaction 0.4 mL of the sample was mixed with 0.6 mL of TBA and 1 mL of sulfuric acid. The resulting mixture was then subjected to incubation at 100 °C for 60 min using a TS-100C Thermo-Shaker (BioSan, Riga, Latvia) [15].

#### 2.4.2. Biochemistry

As a result of increased alcohol consumption following chronic stress exposure, it is of interest to also evaluate biochemical data that characterize liver and kidney function. Plasma samples were used for the determination of the following parameters (units of measure): albumin (g/dL), total protein (g/dL), globulin (g/dL), gamma-glutamyl transferase (U/L), aspartate aminotransferase (U/L), alanine aminotransferase (U/L), alkaline phosphatase (U/L), creatinine (mg/dL), blood urea nitrogen (mg/dL), glucose (mg/dL), Ca^2+^ (mg/dL), phosphate (mg/dL), K^+^ (mmol/L), Na^+^ (mmol/L), total cholesterol (mg/dL). All biochemical parameters were determined by means of a fully automated veterinary clinical chemistry analyzer (Element RC, Heska Corporation, Loveland, CO, USA).

#### 2.4.3. Cortisol Assay

Cortisol levels were measured using a Rat Cortisol Kit (Arbor Assays, K003-H1, Ann Arbor, MI, USA), following the manufacturer’s instructions.

### 2.5. Statistical Analysis

Statistical analyses were performed using GraphPad Prism 9 (GraphPad Software, San Diego, CA, USA). Prior to group comparisons, data normality was evaluated by means of the Kolmogorov–Smirnov test. Depending on the distribution of the data, results are expressed either as mean ± SEM or as median [min–max]. Between-group differences were assessed using the unpaired *t*-test, Mann–Whitney U test, Wilcoxon test or two-way ANOVA with Bonferroni’s post hoc test. A threshold of significance of α = 0.05 was set for all statistical tests.

## 3. Results

### 3.1. Behavioral Tests

#### 3.1.1. Sucrose and Alcohol Preference Test (SPT)

**Figure 2:**
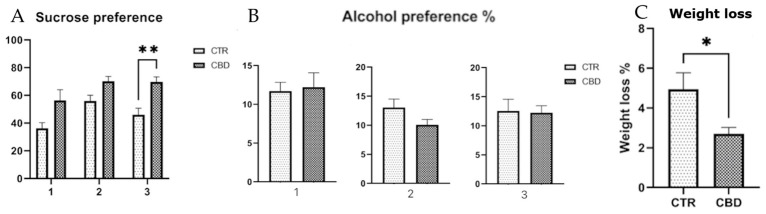
(**A**) Sucrose preference %. Group x time interaction, F(2, 28) = 0.5321, *p* = 0.5932. Time F(1.701, 23.81) = 7.177, *p* = 0.0052. Group F(1, 14) = 19.63, *p* = 0.0006. Two-way ANOVA followed by Bonferroni’s post hoc analysis was employed for sucrose. (**B**) Alcohol preference %. An unpaired *t* test was employed for the first (t(14) = 0.2295, *p* = 0.8218) and third exposure (t(14) = 0.1326, *p* = 0.8964). The Mann–Whitney test was employed for the second exposure. (**C**) Weight loss difference %. An unpaired *t*-test was performed between treatment groups (t(14) = 2.661, *p* = 0.0221). Data are expressed as mean ± SEM. * *p* < 0.05, ** *p* < 0.001.

The SPT finding appear to suggest that rats treated with CBD may exhibit a slightly higher preference for the sucrose solution across the three exposures, which might reflect a relatively preserved responsiveness to rewarding stimuli such as sucrose, as illustrated in Figure 2A. Although no significant difference was observed in alcohol preference, the CBD-treated group consumed a lower volume of alcohol during the second and third exposures as the total volume of liquids consumed decreased. This may have led to an increased percentage preference, as shown in Figure 2B. Additionally, body weight was monitored throughout the experiment, and the CBD-treated group exhibited less weight variation compared to the control group, as shown in Figure 2C.

#### 3.1.2. Open Field Test

**Figure 3:**
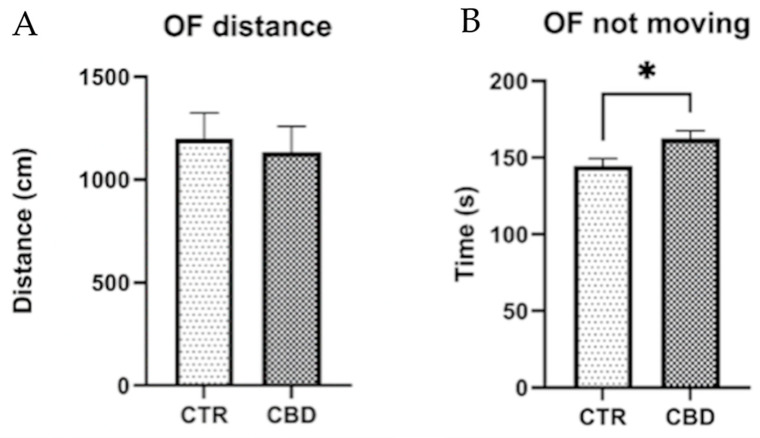
Evaluation of anxiety-related behavior in the OF. (**A**) Total distance traveled throughout the trial. Unpaired *t*-test, t(14) = 0.9470, *p* = 0.3623. (**B**) Duration of freezing behavior recorded during the OF. Unpaired *t*-test, t(14) = 2.551, *p* = 0.0231. The results are presented as the mean ± SEM. * *p* < 0.05.

Analysis of the OF test results revealed no significant differences between the Control and CBD groups with respect to the total distance traveled or the number of entries into the center zone (Figure 3).

#### 3.1.3. Y Maze Test

**Figure 4:**
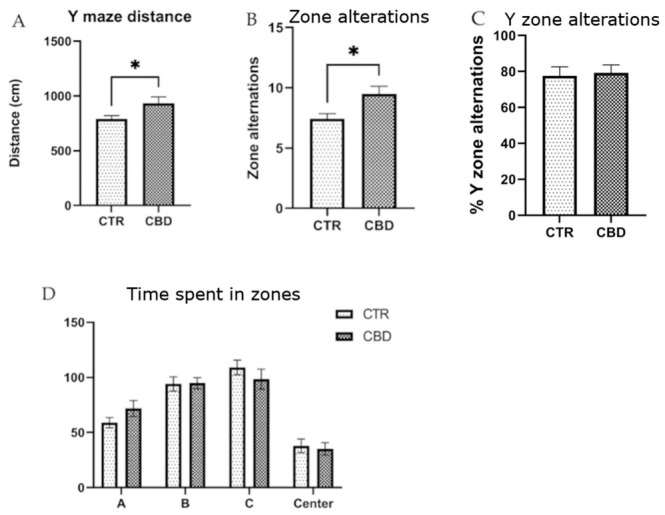
(**A**) Distance traveled during the trial. Unpaired *t*-test, t(12) = 2.195, *p* = 0.0485. (**B**) Number of zone alternations. Unpaired *t*-test, t(12) = 2.647, *p* = 0.0201. (**C**) % Y zone alternations. Unpaired *t*-test, t(12) = 0.4797, *p* = 0.6388. (**D**) Time spent in zones. Interaction F (3, 56) = 1.134, *p* = 0.3433. The results are presented as the mean ± SEM., * *p* < 0.05. A—arm A, B—arm B, C—arm C.

The YM revealed that the CBD-treated group traveled a greater distance compared to the control group, as shown in Figure 4A, and exhibited a higher number of zone alternations (Figure 4B). Minor but non-significant differences were observed in the time spent in different zones (Figure 4D).

#### 3.1.4. Elevated Plus Maze Test

No statistically significant between-group differences were detected in the EPM for any of the parameters examined, including the total distance traveled, the time allocated to the open and closed arms, or the number of entries into the open/closed arms. Head-dipping behavior also did not differ significantly between groups. A trend toward increased rearing duration in the CBD-treated group was observed (Table 1).

#### 3.1.5. Morris Water Maze (MWM)

**Figure 5:**
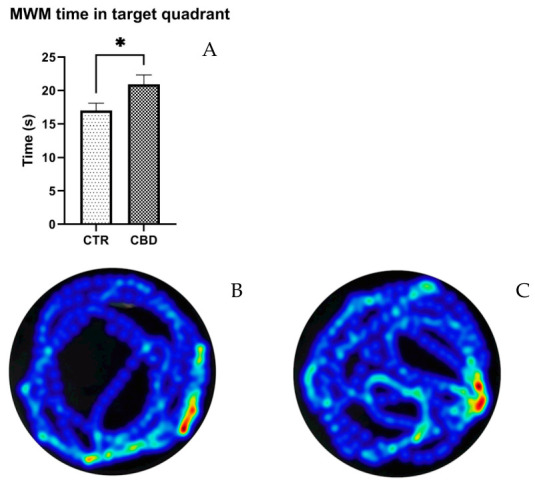
Results of the Morris Water Maze test (MWM) for spatial memory. (**A**) Time in target quadrant. (**B**,**C**) Heatmap of paths. The results are presented as the mean ± SEM. * *p* < 0.05.

As displayed in Figure 5A, CBD-treated animals spent a significantly longer amount of time in the target quadrant than the animals in the CTR group (CBD—20.91 ± 1.434 vs. Control—17 ± 1.090, *p* = 0.0476). None of the other parameters tested statistically differed.

### 3.2. Plasma and Brain Sampling

#### 3.2.1. Oxidative Stress Parameters

**Figure 6:**
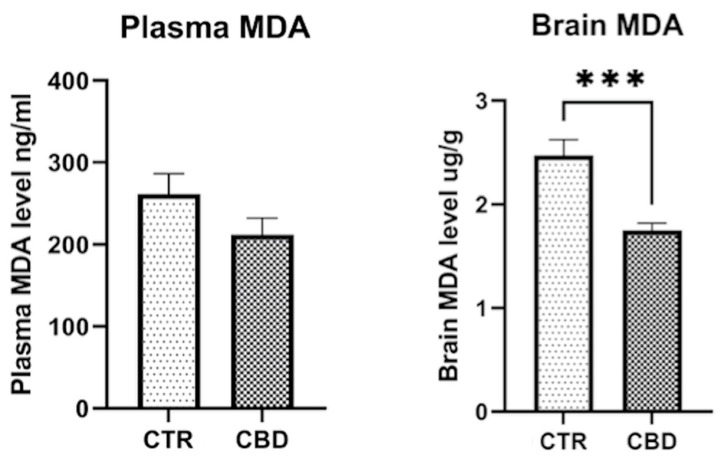
Malondialdehyde (MDA) levels in plasma and brain. The results are presented as the mean ± SEM. *** *p* = 0.0008.

Plasma MDA level showed a slight but not significantly lower level for the CBD group (261 ± 24.97 vs. 212.4 ± 20.41; *p* = 0.1506). The brain MDA level shows significant differences between the two groups (2.471 ± 0.1545 vs. 1.754 ± 0.0672, *p* = 0.0008), as shown in Figure 6.

#### 3.2.2. Biochemistry

Alcohol consumption is known to affect liver enzymes (alanine aminotransferase—ALT and aspartate aminotransferase—AST). The levels of ALT and AST are high, as can be seen in Table 2. However, γ glutamyl transferase, an enzyme specifically used for alcohol consumption, is not affected.

#### 3.2.3. Cortisol Assay

**Figure 7:**
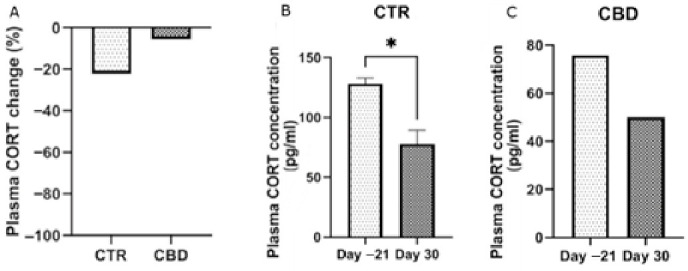
Percentage changes in plasma cortisol (CORT) levels (baseline vs. end of the study) are shown in panel (**A**). They were analyzed using an unpaired *t*-test. Panel (**B**) displays plasma CORT concentrations in the CTR group, and panel (**C**) shows concentrations in the CBD group. The results are presented as the mean ± SEM (**B**) and median (**A**,**C**). A paired *t* test was used for the CTRL group, and the Wilcoxon test was applied for the CBD group. * *p* < 0.05.

By the end of the experimental period, animals subjected to chronic stress displayed a tendency toward elevated cortisol concentrations, as shown in Figure 7A; however, the difference did not reach statistical significance.

## 4. Discussion

To better understand the neurobiological mechanisms underlying this complex interaction, various experimental settings have been developed to study the relationship between depression and chronic stress. Among the most widely used paradigms are chronic mild stress (CMS), social defeat, restraint stress, and other physical or psychological stressors [16]. These experimental models have been validated based on their ability to induce anhedonic, anxiety-like, or depressive-like behaviors in rodents, mirroring key aspects of human depression [17].

Using such models in the context of alcohol research allows the investigation of causal relationships between stress, affective disturbances, and addictive behaviors, as well as the evaluation of pharmacological or behavioral interventions [18]. In this study, we implemented a three-week protocol of unpredictable chronic stress (UCS), during which rats were exposed to randomly scheduled, moderate-intensity stressors.

This type of paradigm mimics the unpredictability and variability of real-life stressors in an ethologically relevant way and is capable of inducing behavioral changes consistent with a depressive phenotype, such as anhedonia, apathy, and avoidance behaviors [19]. Furthermore, it offers a valid framework for studying how chronic stress modulates substance use, particularly alcohol intake, thereby contributing to a deeper understanding of the comorbidity between depressive disorders and addictive behaviors [20].

CBD-treated rats showed a slight tendency toward greater preference for sucrose, suggesting preserved sensitivity to rewarding stimuli. Although their alcohol preference was no different, CBD-treated rats consumed less alcohol in later exposures. Behavioral tests revealed no significant differences, although CBD-treated rats spent more time in the target quadrant. Brain oxidative stress was reduced, while plasma levels showed a non-significant decrease. Liver enzymes (ALT and AST) were elevated due to alcohol exposure, but GGT remained unchanged.

Results obtained in the sucrose preference test indicate that rats treated with CBD maintained a high preference for the sucrose solution throughout the three exposures, suggesting a preservation of the response to rewarding stimuli. This observation is consistent with the literature, which highlights the antidepressant potential of CBD in preclinical models. A study demonstrated that CBD exerts antidepressant effects in animal models, influencing the serotonergic, glutamatergic and endocannabinoid systems, and inducing molecular changes in brain regions associated with depression [21]. It is important to note that all animals used in this study were female, which adds value to the interpretation, as much previous research has been conducted predominantly on male subjects. Some studies suggest that females may respond differently to CBD treatments depending on their estrous cycle, hormonal levels, and stress sensitivity. Resistance to stress-induced anhedonia was observed differentially in females, with females displaying a more pronounced behavioral response compared to males in certain models of chronic stress [22,23]. However, the emergence of significance only at the final exposure in our study should be interpreted with caution. The difference at the third exposure reflects a cumulative treatment effect of CBD, increased sensitivity of the SPT, or both factors. Regarding alcohol consumption, although no statistically significant differences in alcohol preference were observed between the studied groups, CBD-treated rats appeared to show a tendency toward reduced alcohol volume consumption during the second and third exposure. Given that the total volume of liquids consumed was lower, this could potentially contribute to an increased percentage preference. This trend is supported by previous research that has highlighted the ability of CBD to reduce ethanol consumption, motivation to consume, and relapse in animal models. However, other studies have not identified significant effects of CBD on alcohol consumption, suggesting that effects may vary depending on the dose, duration of treatment, and experimental model used [24,25]. The reduction in total fluid intake in the CBD-treated group introduces a methodological confound, as a lower overall drinking volume can inflate the alcohol preference ratio independently of any change in alcohol-seeking motivation, and this confound should be considered when evaluating the apparent tendency toward increased preference in this group. Body weight monitoring throughout the experiment revealed that the CBD-treated group showed smaller weight changes compared to the control group. This result may reflect an anti-stress effect of CBD, preventing catabolism associated with chronic stress. The literature suggests that CBD may influence body weight by reducing inflammation and oxidative stress, as well as by promoting neurogenesis. However, it is important to note that the effects of CBD on body weight may vary depending on the dose, duration of treatment, and individual characteristics of the subjects [26,27,28].

In the Open Field test, no significant differences were observed between groups. CBD-treated females exhibited increased freezing behavior, and therefore these findings do not support a clear anxiolytic effect of CBD. In another study, it was shown that the administration of CBD to females exposed to social isolation led to an increase in the time spent in the center of the arena in the OF test, suggesting an anxiolytic effect. Brtich et al. reported that CBD did not alter OF behavior in rats with inflammatory pain, regardless of sex [29]. The increased freezing behavior observed may reflect heightened vigilance or a stress response, rather than reduced anxiety, suggesting that CBD’s effects on anxiety are highly context-, dose-, and sex-dependent. In contrast, other studies have reported anxiolytic effects in the OF under specific conditions, following social isolation stress in females, suggesting that the anxiolytic potential of CBD may only manifest in the presence of a specific stressor or during a particular exposure window [30]. In the present study, no statistical difference was observed in the EPM test, except for rearing, which suggests exploratory behavior in the vertical area. In another study, CBD reduced anxious behaviors in the Elevated Plus Maze test in females [31,32]. The increase in rearing observed in our study may represent a specific component of exploratory behavior rather than a generalized reduction in anxiety.

The Y maze test reflects the ability to retain information about the immediately previous environment by measuring the tendency to explore a new arm of the maze at the expense of those already explored. Thus, the distance traveled by the rats treated with CBD was greater than in the CTR group and a greater number of alternations was also observed, suggesting possible memory retention. In a study conducted by Contreras A et al., intermittent alcohol administration affected spatial memory in Y maze [33]. In another study, CBD exposure was shown not to influence memory in Y maze; however, sex interactions were observed [34]. Razavi et al. showed that CBD use can improve the disruption of spatial memory in the abstinence phase after methamphetamine exposure [35].

To evaluate cognitive functions, we employed the MWM test, in which the CBD group showed improved performance compared to controls. Although all animals successfully located the platform, those in the CBD group spent significantly more time in the target quadrant. A recent study demonstrated that chronic administration of CBD for four weeks in middle-aged female rats subjected to social isolation significantly improved spatial memory, evidenced by their reduced latency to find the platform and increased time spent in the target quadrant [36]. Similar results were also reported in male mice exposed to social isolation, where CBD treatment prevented memory impairment [37]. However, a study conducted on mice showed no influence on spatial memory on MWM test [38], supporting the interpretation that the memory-enhancing effects of CBD may depend on pre-existing cognitive vulnerability, stress or isolation, rather than a direct effect.

For this study, we also investigated the effect of CBD on MDA in plasma and the brain as a marker of oxidative stress. The potential antioxidant effect of CBD was observed as a reduction in MDA levels; however, this decrease reached statistical significance only in the brain, while plasma MDA showed no significant change (*p* = 0.1506), highlighting that the effect may be limited to the CNS. While our brain MDA results align with those in [39], the absence of a significant plasma effect in our study diverges from the same study. This difference may reflect dose and duration effects. Another study found that 90 days of CBD exposure significantly reduced plasma MDA in rats, suggesting that the dose and duration may affect MDA levels [40]. A study observed that CBD increased cardiac and plasma lipid peroxidation in normotensive rats while reducing it in hypertensive animals, suggesting that the antioxidant effect on peripheral oxidative stress is dependent on context [41].

In our study, female rats in the control group that consumed alcohol showed significantly increased levels of the liver enzymes ALT (alanine aminotransferase) and AST (aspartate aminotransferase) compared to the CBD-treated group. While AST and ALT levels were lower in the CBD group, this likely reflects their reduced alcohol consumption rather than a direct hepatoprotective effect of CBD. The absence of differences in GGT, a more specific marker of alcohol-related liver injury, further suggests that these enzyme changes may be driven primarily by differences in alcohol intake. One study found that CBD protects hepatocytes from steatosis induced by alcohol consumption [40]. Involved mechanisms include prevention of oxidative stress and increased autophagy; both are considered essential processes in maintaining liver homeostasis. These results suggest that CBD can prevent lipid accumulation in the liver and subsequent deterioration of liver function [40]. Research has shown that CBD can attenuate ethanol-induced liver toxicity by inhibiting the JNK/ERK/MAPK signaling pathways, thereby reducing inflammation and oxidative stress in the liver. This mechanism may contribute to protecting hepatocytes from alcohol-induced injury [41,42,43,44].

After chronic exposure to stress, the CTR group showed lower cortisol levels by the end compared to the CBD group, although this difference was not statistically significant. Cortisol secretion is influenced by multiple factors, and chronic stress can lead to variable responses [39]. While acute stress typically increases cortisol, prolonged stress may alter hypothalamic–pituitary–adrenal axis function, although our results do not provide clear evidence for such dysregulation.

The study has several important limitations that must be highlighted when interpreting the results. We included only female subjects, which limits the generalizability of the results. In addition, no measurements of serotonin and dopamine, essential neurotransmitters in the modulation of anxiety and depression, were performed, nor were histological studies performed to evaluate the structural impact of the treatment. Also, the administration of CBD was performed with a single dose type. Only two groups were included with no non-stressed control group, making it difficult to determine whether the observed behavioral and biochemical changes were due to stress, alcohol, CBD, or their interaction. The group size was small, limiting the statistical power, and the estrous cycle was not monitored. The alcohol exposure protocol was brief and intermittent. Assessment of oxidative stress relied solely on MDA and behavioral testing was carried out with only partial blinding. Mechanistic insight was limited, with no evaluation of specific neurobiological pathways, and CBD exposure was not confirmed by pharmacokinetic studies, with administration through food potentially adding variability. These factors highlight the need for caution in interpreting the findings and underline the need for future studies to confirm the observations.

Both the limitations and our results plead for further studies that could explore the role of CBD in the chronic, unpredicted stress model of depression.

## 5. Conclusions

The CBD-treated group showed a trend toward lower alcohol consumption compared to the control group, although this difference did not reach statistical significance, as well as an increased preference for sucrose solution. The MWM test revealed a protective effect on spatial memory, while MDA measurements revealed the potential central antioxidant effect of CBD.

## Figures and Tables

**Table 1:** Parameters of the Elevated Plus Maze test.

Parameter	CTR	CBD	*p* Value
Distance	953.8 ± 74.49	1093 ± 80.48	t(12) = 1.226 *p* = 0.2420
Time in open arms	32.20 ± 6.048	32.62 ± 8.105	t(12) = 0.4124 *p* = 0.9678
Time in closed arms	178.6 ± 13.38	184.9 ± 20.18	t(12) = 0.2584 *p* = 0.8005
Entries in open arms	26.13 ± 3.87	24.38 ± 4.807	t(12) = 0.2836 *p* = 0.7809
Entries in closed arms	31.63 ± 4.788	27.71 ± 4.275	t(12) = 0.5578 *p* = 0.5578
Head dipping	8.857 ± 1.262	10.43 ± 1.875	t(12) = 0.6952 *p* = 0.5
Rearing	17.18 ± 1.670	27.87 ± 5.460	t(12) = 2.117 *p* = 0.0558 *
Freezing	150.6 ± 12.90	135.9 ± 9.792	t(12) = 0.9087 *p* = 0.3814

* The result is close to statistical significance. The results are presented as the mean ± SEM.

**Table 2:** Biochemical parameters.

Biochemical Parameter	Group	Mean ± SEM	*p* Value
Albumin	CTR	4.133 ± 0.05489	0.7985
	CBD	4.075 ± 0.1333	
Total protein	CTR	6.563 ± 0.09625	0.8098
	CBD	6.513 ± 0.1797	
Globulin	CTR	2.4 ± 0.08	0.6534
	CBD	2.5 ± 0.203	
GGT	CTR	27.75 ± 5.987	0.5271
	CBD	24.50 ± 5.217	
AST	CTR	329.6 ± 71.76	0.0231 *
	CBD	151 ± 17.44	
ALT	CTR	105.1 ± 8.523	0.0347 *
	CBD	81.5 ± 5.713	
ALP	CTR	213.6 ± 16.17	0.3937
	CBD	244.6 ± 31.3	
Creatinine	CTR	0.4750 ± 0.0411	0.9551
	CBD	0.4875 ± 0.0548	
BUN	CTR	19.99 ± 0.9290	0.4219
	CBD	18.85 ± 1.009	
Glucose	CTR	179.6 ± 6.426	0.1499
	CBD	165.6 ± 6.440	
Cholesterol	CTR	87.62 ± 7.690	0.8072
	CBD	89.98 ± 5.598	
Ca^2+^	CTR	9.688 ± 0.09675	0.7537
	CBD	9.739 ± 0.1277	
PHOS	CTR	4.386 ± 0.2449	0.1724
	CBD	4.916 ± 0.2755	
Na^+^	CTR	148.5 ± 0.1784	0.6279
	CBD	148.6 ± 0.2217	
K^+^	CTR	5.6 ± 0.1813	0.6240
	CBD	5.749 ± 0.2125	

GGT—γ glutamyl transferase; AST—aspartate aminotransferase; ALT—alanine aminotransferase; ALP—alkaline phosphatase; BUN—blood urea nitrogen; PHOS—phosphate; Mann–Whitney U: GGT, creatinine, Na^+^. * *p* < 0.05.

## Data Availability

The original contributions presented in this study are included in the article. Further inquiries can be directed to the corresponding author.

## Abbreviations

The following abbreviations are used in this manuscript:

ALP	Alkaline phosphatase
ALT	Alanine aminotransferase
AST	Aspartate aminotransferase
BUN	Blood urea nitrogen
CBD	Cannabidiol
CMS	Chronic mild stress
CORT	Cortisol
CTR	Control
EPM	Elevated plus maze
GGT	γ-glutamyl transferase
HPA	Hypothalamic–pituitary–adrenal
MDA	Malondialdehyde
MWM	Morris water maze
OF	Open field
PHOS	Phosphate
ROS	Reactive oxygen species
SPT	Sucrose preference test
UCS	Unpredictable chronic stress
WHO	World Health Organization
YM	Y maze
